# Corrosion and Wear Behavior of TiO_2_/TiN Duplex Coatings on Titanium by Plasma Electrolytic Oxidation and Gas Nitriding

**DOI:** 10.3390/ma15238300

**Published:** 2022-11-22

**Authors:** Hassan Bakhtiari-Zamani, Ehsan Saebnoori, Hamid Reza Bakhsheshi-Rad, Filippo Berto

**Affiliations:** 1 Advanced Materials Research Center, Department of Materials Engineering, Najafabad Branch, Islamic Azad University, Najafabad, Iran; 2 Department of Mechanical and Industrial Engineering, Norwegian University of Science and Technology, 7491 Trondheim, Norway

**Keywords:** plasma electrolytic oxidation (PEO), gas nitriding, adhesion, corrosion, tribological properties

## Abstract

In this study, corrosion and wear behavior of three kinds of coatings by two processes, namely, plasma electrolytic oxidation (PEO) coatings (Ti/TiO_2_), gas nitriding coating (Ti/TiN), and the duplex coating (Ti/TiO_2_-N) by combination of PEO and gas nitriding methods were systematically investigated. X-ray diffraction tests, field-emission scanning electron microscopy, and adhesion tests are employed for the coating characterization, along with the wear and electrochemical test for evaluating the corrosion and tribological properties. The morphology and structure of the coating consist of micro-cavities known as the pancake structure on the surface. The electrolytic plasma oxidation process produces a typical annealing behavior with a low friction coefficient based on the wear test. The coating consists of nitride and nitrate/oxides titanium for nitrided samples. The surface morphology of nitrided oxide titanium coating shows a slight change in the size of the crystals and the diameter of the cavities due to the influence of nitrogen in the titanium oxide coating. The tribological behavior of the coatings showed that the wear resistance of the duplex coating (Ti/TiO_2_-N) and Ti/TiO_2_ coatings is significantly higher compared to Ti/TiN coatings and uncoated Ti samples. The polarization resistance of the Ti/TiO_2_-N and Ti/TiO_2_ coatings was 632.2 and 1451.9 kΩ cm^2^, respectively. These values are considerably greater than that of the uncoated Ti (135.9 kΩ cm^2^). Likewise, impedance showed that the Ti/TiO_2_-N and Ti/TiO_2_ coatings demonstrate higher charge transfer resistance than that of other samples due to better insulating behavior and denser structure.

## 1. Introduction

Titanium and its alloys have excellent properties, such as density and elastic modulus, high melting point, low ratio of strength to weight, excellent corrosion resistance, biocompatibility, high resistance to creep and fatigue, and appropriate mechanical and nonmagnetic properties [1,2]. However, titanium has some tribological weakness that limits its applications. These limitations are high friction coefficient, low adhesion and abrasion wear resistance, and low hardness. These drawbacks can be attributed to titanium’s crystalline and electronic structure [3]. Different methods have been employed to improve the surface properties and increase corrosion resistance, primarily based on applying a coating layer or converting the surface to a coating, such as surface oxidation, PVD/CVD coatings, ion implantation, sol–gel, etc. [1].

TiO_2_ films can be formed on a titanium substrate by different methods, such as surface oxidation, magnetron sputtering, sol–gel, physical vapor deposition (PVD), chemical vapor deposition (CVD), and ion implantation [4]. The mentioned techniques are costly and, in the case of ion implantation, they is very thin, and for CVD, it is not adhesive enough. Therefore, the oxidation method can be considered one of the most important techniques for titanium surface modification. In recent developments, high-voltage anodic oxidation has been employed at temperatures close to ambient temperature to form crystalline TiO_2,_ including rutile and anatase. These new electrochemical methods are called plasma electrolytic oxidation (PEO) [1,5]. In recent years, more attention to light alloys for transportation and aerospace applications has made the PEO an effective coating technology to protect against corrosion and wear light alloys [6,7,8,9]. PEO also has drawn considerable attention due to the fact that it can substantially boost corrosion and wear resistance in Ti alloy substrates owing to the generation of oxide layers on the top of the substrate surface. The double-layer coatings formed by these methods can significantly reduce the friction coefficient and improve wear resistance [10,11,12,13,14]. In this respect, the coatings are generally composed of two layers: a PEO ceramic inner layer and a solid outer lubricating layer; when the outer layer is worn, the tribological properties are returned to the initial level [15,16,17,18,19].

Gas nitriding is a commonly used method for engineering applications due to the formation of hard layers on the surface. The main advantage of this process is that it does not need any expensive equipment and can be applied to parts with different geometries. The formation of a nitride layer on titanium and titanium alloys is a complex process consisting of several reactions that coincide with the interface between gas and metal and surface layers [20,21,22]. Generally, the microstructure and the tendency for grain growth in titanium and its alloys depend on temperature and time in the nitriding process [21]. Nitrogen incorporated into titanium oxide from ammonia gas is partially doped to the surface layer at less than 600 °C. If the nitriding temperature exceeds 600 °C, the nitrogen addition in titanium oxide increases and reduces the oxygen content of the coating to the extent that the non-stoichiometric composition of TiO_x_N_y_ is converted into the stable TiN [22].

The effect of composite TiO_2_-Ti and TiO_2_-TiN coating on NiTi surfaces has been investigated by Maleki et al. [23]. In this study, titanium oxide was coated on a NiTi by the electrophoretic method; then, the samples were placed in two separate furnaces (Ar and N_2_) at 1000 °C. The nitriding led to joining particles (sintering) and reduced the contractions due to the increase in the volume of the nitride phase. According to Alsaran’s research, the duplex coating has three levels of surface oxide, mixed and diffusive layers. They showed that the oxide layer (8–10 μm) had a much higher hardness than the composite and diffusive layer (thickness of 90–110 μm). Resultingly, the hardness of the duplex coating is much more than simplex coatings. Moreover, it is reported that the friction coefficient and wear rate was less for coating duplex than for uncoated samples, oxide coatings, and nitride coatings, which is due to the presence of hard and stable phases, such as rutile and TiN [24]. Up until now, a number of investigations have been carried out [10,11,12,13,14,15,16,17,18,19,20,21,22] on PEO coatings on Ti and its alloys with the purpose of enhancing their corrosion resistance and wear performance of the substrate. Nevertheless, to the best of our knowledge, a few investigations are attainable in the literature concerning TiO_2_/TiN duplex coatings on titanium by a combination of plasma electrolytic oxidation and gas nitriding methods. Hence, the motivation of this study is to investigate the corrosion and abrasion behavior of TiO_2_-N duplex coatings formed by plasma electrolytic oxidation and gas nitriding.

## 2. Experimental Procedure

The commercially pure titanium grade 2 from TIMET, Germany was used for this study. The 13 × 13 mm samples were cut from a sheet with a thickness of 3 mm and then were abraded by SiC sandpaper up to 1200 grit. Next, the organic contaminants were ultrasonically removed in acetone for 10 min, rinsed with distilled water, and dried by hot air. The chemical composition of titanium as the substrate is presented in the supporting information (Table S1). The coating process was firstly performed by plasma electrolytic oxidation to form titanium oxide. In this method, the titanium specimen acted as an anode (positive pole), a stainless steel sheet was used as the cathode (negative pole), and 0.2 M sodium carbonate with 5 g/L sodium hydroxide aqueous solution was used as the electrolyte according to Ref [13]. The PEO coating for preparation of TiO_2_ film on top of the Ti substrate was carried out under frequency of 1500 Hz and current density of 100 mA/cm^2^. In this regard, the duty cycle and process time were 10 % and 10 min, respectively, under unipolar pulsed current. The coated specimens were rinsed in deionized water and subsequently dried in hot airflows. Afterward, the supplemental coating operation was performed in the second step by gas nitriding in a tube furnace with passing N_2_ gas (99.99%). At this stage, a titanium specimen (with the name of Ti/TiN) and a TiO_2_ coated titanium sample by PEO (with the name of Ti/TiO_2_-N) were placed inside the furnace. In order to increase adhesion and density, and also to reduce the porosity of the coatings, the nitriding process took place at 1000 °C and 1.5 bar nitrogen pressure for 6 h, according to Ref [20]. The heating rate of 8 °C/min was set for the first 2 h to reach 1000 °C. Then, the samples were kept at the temperature of 4 h and finally cooled in the furnace. The nitrogen gas flow is maintained during heating and cooling.

X-ray diffraction on the coated samples was carried out to identify the phases by PHILIPS PW 1730 X-ray diffractometer equipped with Cu-Kα radiation (40 kV, 30 mA). Fourier transform infrared spectroscopy (FT-IR) was also employed for determining the structures of molecules and chemical species. The Ti/TiO_2_-N sample FTIR spectrum was taken using a Thermo Fisher Scientific (Madison, WI, USA) between 500 cm^–1^ and 4000 cm^–1^. Surface and cross-sectional images were taken by a scanning electron microscope (Leo, 435 VP, Cambridge, UK), and, for higher magnification, a FESEM (TESCAN, Mira 3-XMU, Brno, Czech Republic) was employed. The corrosion behavior of the samples was evaluated in a 3.5% sodium chloride aqueous solution at ambient temperature by a PARSTAT 2273 potentiostat. The samples were tested for electrochemical potential stability within the solution for 60 min before the electrochemical tests, and their open circuit potentials (E_OCP_) were measured. A standard three-electrode electrochemical setup consists of a saturated calomel as a reference electrode, graphite as an auxiliary electrode, and the sample as a working electrode used for electrochemical measurements. For the polarization test, the potential scanning rate was set at 1 mV/s, started from 0.25 volts below the E_OCP,_ and continuing to 2 volts above the E_OCP_. The EIS test was performed in the range of 10 mHz to 100 kHz at the E_OCP_ with an AC potential of 10 mV amplitude. The Koopa KM3 micro-hardness device (Vickers) was used to measure the hardness of the coating according to the ASTM E 384 standard, which was calculated from the size of an impression produced under 500 g load by a pyramid-shaped diamond indenter for 10 s. To study the tribological properties of the coatings, such as friction coefficient, weight loss, and abrasion rate, a non-lubricated pin on disk wear test (ASTM G 99) was used. In this test, the samples were placed as the disk, and an AISI 52100 (hardness 64 Rockwell C) with a diameter of 5 mm and a length of 50 mm was used as the abrasive pin. The TSN-WTC 02 was employed for the wear test under laboratory conditions (T = 25 ± 2 °C and 21% humidity) with a load of 2 N and a speed of 0.05 m/s with a distance of 100 m.

## 3. Results and Discussion

### 3.1. Plasma Discharge Characteristics

**Figure 1:**
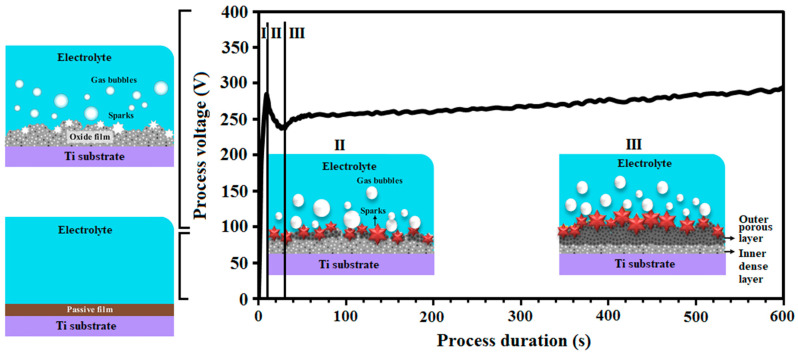
The V-t chart for the PEO process.

Figure 1 shows the voltage-time diagram for the coating process during electrolytic plasma oxidation. It is divided into three regions to understand the behavior of voltage changes better. In Region I, the voltage linearly and steeply increases, and the average voltage increase rate is 32 V/s. At this stage, with the voltage rising, conventional anodizing occurs [25] so that a very thin oxide film with a high resistivity is formed on the surface. The amount of voltage increase for the sample was 285 V in around 9 s. In region II, the graph shows a lessening trend, with a decrease of 17%, indicating the failure of the formed TiO_2_ oxide in part I. In other words, the potential reduction in this region is due to the effect of ions and charge transfer simultaneously, while in area I, the ascending trend is the consequence of the ionic current [26]. The voltage drop for this sample occurred within 20 s from 211 V to 285 V. The process of sparking occurs in this region. In region III, the slop is positive, and the potential values increase noticeably. The rise in voltage starts at 31 s, and the voltage changes from 237 to 295 volts. In this region, the micro-arc color transforms from white to orange over time and, in the end, the number of sparks decreases, and their color becomes orange. The whole surface is coated with the TiO_2_ ceramic layer, and the voltage curve gradually reaches a steady state. The voltage fluctuations are also observed due to several micro arcs and, at the end of this stage, only a few sparks happen, confirming this region’s process.

### 3.2. Crystal Structure and Coating Morphology

**Figure 2:**
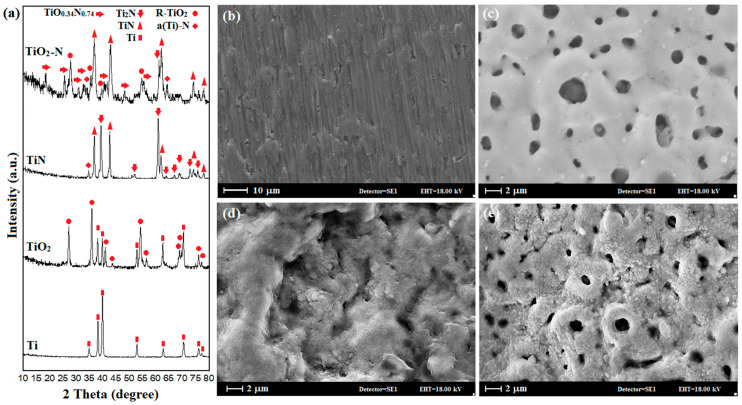
(**a**) The XRD patterns and SEM images of (**b**) bare sample, (**c**) TiO_2_ coated by PEO method, (**d**) TiN coated by gas nitriding, and (**e**) nitrided TiO_2_ coated sample (duplex coating).

Figure 2a shows X-ray diffraction patterns after PEO, gas nitriding, and the uncoated sample. Table 1 also shows the Ti/TiO_2_, Ti/TiN, and Ti/TiO_2_-N coatings’ crystallographic properties. The X-ray diffraction pattern of the TiO_2_ sample, which PEO forms on the Titanium substrate, consists of 97.5% tetragonal crystalline rutile titanium oxide. The weight percentages of the oxides were obtained from the peak intensity in the XRD patterns and calculated by Equations (1) and (2) [27,28].

(1) $$X_{R}=\frac{1}{1+0.8\left(\frac{I_{A}}{I_{R}}\right)}$$

(2) $$X_{A}=1-X_{R}\;\;\mathrm{or}\;\;X_{A}=\frac{1}{1+1.26\left(\frac{I_{R}}{I_{A}}\right)}$$

In the above equations, X_R_ and X_A_ are the weight percentage of the rutile and anatase. Furthermore, The I_R_ and I_A_ are anatase (101) and rutile (110) peak intensities. Some titanium peaks are observed in this pattern due to the X-ray penetration into the substrate via the microcavities or the coating. The coating formation conditions for this sample were such that the anatase phase is deficient; this is due to the heating effects of plasma micro-arcs during coating formation and having a temperature above 673 K. In other words, the dominant mechanism of oxide layer formation is thermal oxidation [29,30].

The XRD pattern of the Ti/TiN sample is associated with the gas nitrided titanium. The flow of nitrogen gas at high temperatures above the titanium surface leads to nitrogen adsorption, penetration to the surface, and the formation of a nitride film. In other words, the reaction of nitrogen with the substrate forms a nitride layer consisting mainly of the cubic titanium nitrides, such as TiN and Ti_2_N, followed by the inner diffusive region in the HCP α-Ti solid solution phase. It has been seen that the patterns of gas nitrided samples, TiN, Ti_2_N, and, somehow, α-(N)-Ti peaks, overlap with titanium peaks due to the presence of α-Ti solid solution doped by nitrogen [31,32]. In the Ti/TiO_2_-N sample, more than the abovementioned phases, the TiO_0.34_N_0.74_ phase with monoclinic structure along with rutile TiO_2_ is also detected. The TiO_0.34_N_0.74_ phase is formed due to the replacement of nitrogen atoms with some oxygen in the TiO_2_ structure, a non-stoichiometric compound. The obtained peaks from this sample are wider than the other specimens, suggesting a smaller crystallite size. In this study, the Scherrer equation [22], with an index peak of (111) at 37.55 degrees, was used to determine the crystallite size of Ti/TiN and Ti/TiO_2_-N samples. The results showed that the size of the crystallites decreased from 24.3 to 17 nm for Ti/TiN to Ti/TiO_2_-N samples. The apparent color of the sample depends on the process parameters and the formation of nitride phases [33,34], while in uniform surface morphology with golden brown color, the formation of TiN can be observed [35].

Figure 2b–e shows the SEM images of the surface of bare and coated samples by PEO and gas nitriding. The uncoated titanium sample (Ti) surface in Figure 2b shows parallel lines originating from the abrasion by sandpaper. The morphology of the Ti/TiO_2_ sample (Figure 2c) consists of grains of different sizes and micro-cavities on these grains (pancake-like structure) [1,25]. The top surface of this coating has a distinct appearance: the surface consists of a large number of pores of various sizes; some of these pores are surrounded by relatively smooth re-solidified pseudo circle areas. When the discharge channels cool, the reaction products in the channel walls precipitate near the discharge channels, thus forming closed holes similar to the volcano [36,37]. The presence of surface cracks in the ceramic coating produced by the PEO method is due to the internal stresses of the coating, the contraction of the melt pool produced by electrical discharge, and the difference between the produced phases in the outer and inner layer of the coating [38]. Another reason for micro-cracks formation is the thermal stress caused by rapid cooling. The TiN sample (Figure 2d) looks dense, and the nitrated layer is formed on the surface. The sample has some defects, including small pits and micro-cracks on the surface. Mismatching of thermal contraction between the formed phases can lead to residual thermal stress during cooling down from nitriding temperature to room temperature. On the other hand, deficiencies in nitride and oxide structures can cause residual stress [39]. Generally, high residual stress can cause cracking in the coating and morphological deformation. It is worth noting that increased penetration of titanium ions through the grain boundary and reaction with nitrogen atoms can cause defects such as protrusions and small cavities on the nitrated surface of the titanium. The Ti/TiO_2_-N sample (Figure 2e) is similar to that of the Ti/TiO_2_ sample structure, except for the spherical cavities present in the so-called cauliflower structure. The Ti/TiO_2_-N sample cavities are smaller and sometimes more faded than the Ti/TiO_2_ sample due to the penetration of nitrogen into the titanium oxide structure (pore diameter decreased from 4.4 microns to 2.8 microns). The nitriding process’s thermal energy results in the re-crystallization of the TiO_2_ phase of the surface coating.

**Figure 3:**
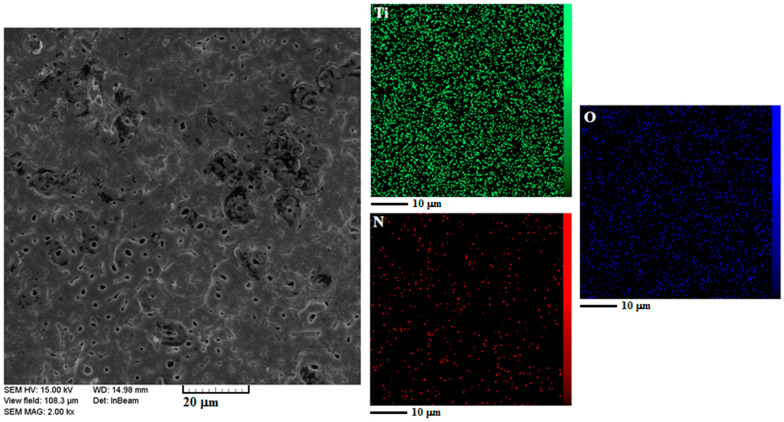
The elemental distribution map of Ti/TiO_2_-N sample surface.

Figure 3 shows the element mapping analysis of the Ti/TiO_2_-N duplex coating surface. Since in this study, the process of nitrogen entry into titanium oxide was considered, the distribution of elements on the surface of coating was investigated, and the results showed that nitrogen penetrated evenly between titanium oxide and titanium metal.

**Figure 4:**
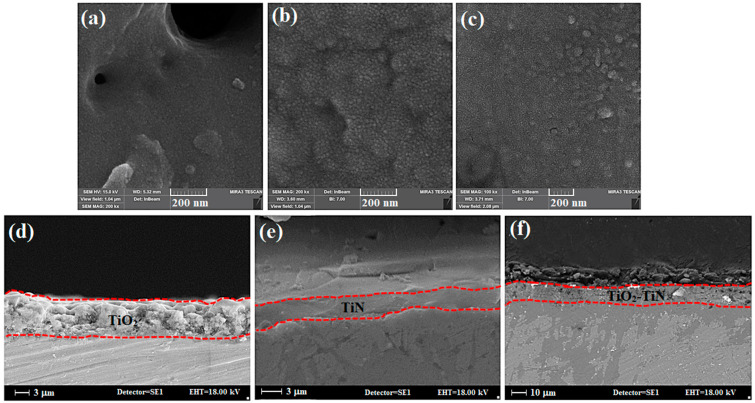
FE-SEM images of the surface morphology and cross-sectional images of the coated samples. (**a**,**d**) Ti/TiO_2_, (**b**,**e**) Ti/TiN, and (**c**,**f**) Ti/TiO_2_-N.

Figure 4a–c shows the FE-SEM images of the surface morphology of the samples coated with PEO and gas nitriding. The average crystallite sizes for Ti/TiO_2_, Ti/TiN, and Ti/TiO_2_-N samples were 18.9, 14.1, and 13.7 nm, respectively. In the Ti/TiO_2_ sample, the formed grains could not grow, and the size of the crystallites decreases. Since temperature is the most crucial factor in reducing grain size for nitrated samples (Ti/TiN and Ti/TiO_2_-N), gas nitriding and nitrogen penetration at titanium surface and oxide structure are performed. Due to its smaller atomic radius and its replacement with oxygen, titanium did not allow the TiO_2_ grains to grow, and the size of the crystallites is decreased. Cross-sectional images of Ti/TiO_2_, Ti/TiN, and Ti/TiO_2_-N coated samples are shown in Figure 4d–f. The layer thicknesses of the samples are 7.9, 3.3 (3.3 μm TiN & Ti_2_N layer and about 25 μm diffusion zone), and 8.7 μm, respectively (as shown with red dotted lines). It can be seen that filling pores and porosities are observed after the gas nitriding process on Ti/TiO_2_ coating by plasma electrolytic oxidation, which confirms the nitrogen diffusion into the titanium oxide coating.

### 3.3. Wear Property and Micro-Hardness of the Coating

**Figure 5:**
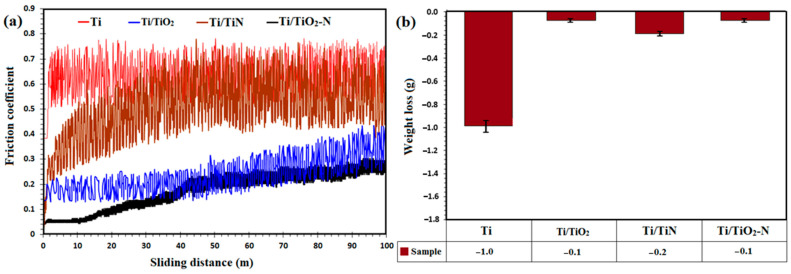
(**a**) The diagrams for friction coefficient changes versus slide distance and (**b**) weight loss for uncoated Ti alloy and Ti/TiO_2_, Ti/TiN, and Ti/TiO_2_-N coated samples.

Figure 5a shows the friction coefficient diagram of the uncoated titanium and Ti/TiN, Ti/TiO_2_, and Ti/TiO_2_-N duplex coatings for a distance of 100 m with an applied load of 2 N. The coefficient friction of the uncoated specimen is about 0.65, on average, over a distance of 100 m. It can be said that the highest coefficient of friction and the minor wear resistance among all specimens is Ti. The coefficient friction in the resulting diagrams can be divided into two dynamic and steady stable regions. In some cases, the coefficient friction reaches a steady-state immediately after the wear process starts from the starting point and remains there until the end. While the dynamic and incremental part can sometimes be attributed to surface roughness, after removing some roughness by the pin, and after a real contact level is formed, the coefficient friction reaches a constant value and remains at the same point. In the steady-state region, there are two low-amplitude and wide-amplitude oscillations. Low amplitude oscillations may be due to intermittent local breaks in the surface parts of the coating. Wide fluctuations can be due to a new phase during abrasion with a different coefficient of friction to the pin.

According to the above explanation, the friction coefficient diagrams of the coatings can be described. In the Ti/TiO_2_ sample, the average coefficient of friction is about 0.2. The oscillations in this sample are as follows, consisting of two steps: a dynamic zone that lasts up to 44 m with a low oscillation amplitude due to the roughness of the coating and the transfer of rough surface, or outer porous layer, to the soft inner surface of the coating. The steady-state zone, which ranges from 44 m with low oscillation amplitude to the end, is associated with the accumulation of abrasive particles. In this diagram, we sometimes see sudden fluctuations and valleys that may be due to removing a piece of ceramic coating.

In the Ti/TiN sample, the amplitude of friction coefficient variations (0.25 to 0.65) is similar to that of the uncoated sample, with an average of about 0.5. The dynamic zone is short and enters the steady-state zone over a distance of 2 m. In other words, the test results have a tiny dynamic area, indicating that the roughness of the coating was low and that the surface was almost smooth during friction. The oscillations have an increasing trend along with the broader range due to new phases during the wear process. Additionally, in this case, due to the free energy required for Ti/TiO_2_ formation during wear, the TiN coating lubricates because the titanium oxide phase is easy to crack and cut before adhesion [40]. In the Ti/TiO_2_-N sample, the range of friction coefficient changes (0.15 to 0.35) is similar to that of Ti/TiO_2_, and the average coefficient of friction can be reported to be about 0.25. The steady-state zone mainly can be seen for this sample, while after a few sliding distances after starting point and reaching the steady-state zone, the trend changes slowly with a slight upward slope. The amplitude of the oscillations in this sample is relatively low, and it can be said that there are very few surface breaks due to abrasive wear.

According to the weight change diagram in Figure 5b, it can be found that the highest and the lowest weight loss are for Ti and Ti/TiO_2_-N, respectively. The weight loss in the uncoated sample was low due to wear resistance, while the weight of the slider pin increased due to the retention of titanium particles removed from the surface and accumulation on the pin. The weight loss has a slight difference between the coated specimens, which indicates an increased wear resistance.

It is noticeable that the weight loss occurs in the pin for the coated samples due to increasing the wear resistance of the coating, and more wear takes place for the pin. In the case of Ti/TiO_2_-N coating, it can be said that low weight loss (close to zero) does not mean that there is no weight loss at all, but rather a minimal amount of weight loss with weight gain due to compensating some weight loss by the residual pin-worn iron particles on the sample surface.

These results indicate a high increase in the wear resistance of nitrided samples. Another notable result is the weight loss of steel pins, which can conclude that the nitride coatings are resistant to the steel surface and severely abrade this surface. The Ti/TiO_2_-N coating presented a lower wear loss and friction coefficient than that of the bare titanium substrate. It is shown that in the XRD results and surface hardness, the hardness of the formed rutile layer, which is subjected to the nitriding process, is very high and reduces the wear rate. Besides, the increased wear capacity of the nitrated specimen increased the wear resistance because the friction was limited in the elastic range [24].

**Figure 6:**
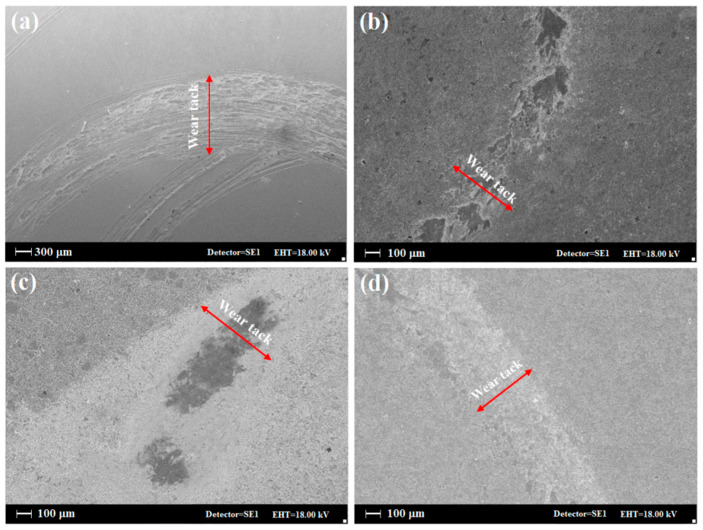
Images of wear sites of samples: (**a**) uncoated Ti, (**b**) Ti/TiO_2_, (**c**) Ti/TiN, and (**d**) Ti/TiO_2_-N.

Figure 6 shows the uncoated Ti and coated samples’ images after the wear test at various magnifications, which confirm the friction test results. It is shown in the figure that the titanium wear mechanism is a combination of the abrasive and adhesive wear mechanism, which may be caused neither by plastic deformation nor by adhesion, but by pin abrasion. It should be considered that the pin gets rough due to the abrasion process and partially causes abrasive wear. The adhesion mechanism increases the width of the wear effect on the titanium surface, and the particles are adhesively removed from the surface during the test. On the other hand, for coated specimens, the amount and scars of wear are insignificant. Generally, due to the specific surface morphology and the presence of bumps and potholes, a completely different mechanism prevails. The surface is not worn, and the surface bumps are compressed in the course of the test. If any particles are removed from the bumps, they are included in the surface potholes. Additionally, because titanium nitride is harder than pins, particles from the pin can be embedded in surface pits associated with a reduction in friction coefficient.

**Figure 7:**
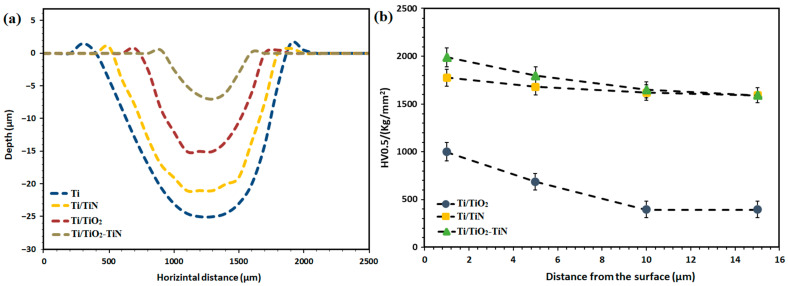
(**a**) Linear profiles of wear depth of uncoated and coated samples, and (**b**) Depth micro-hardness profile obtained on the coated samples by PEO and gas nitriding.

Conclusively, for the coated samples, it can be said that the nitriding process has increased the wear resistance of titanium; in other words, the wear resistance of Ti/TiO_2_-N coating regarding Ti/TiO_2_ sample has increased, which is attributed to the strengthening effect and the reduction of the friction coefficient. Hardness and friction coefficient are two factors that influence the wear resistance of coatings. Figure 7a shows the linear profiles of the wear depth of the specimens by the weight loss and images of wear tracks. According to this graph, it is found that the bare Ti sample with 25 μm has the highest wear depth, while the Ti/TiO_2_-N sample has the lowest wear depth (6 μm), implying the higher wear resistance provided by the creation of the duplex coating on the surface of the titanium.

Figure 7b shows the microhardness profiles along the cross-section (as a function of depth) for the coated samples. The Ti/TiO_2_-N sample has the highest surface hardness. For Ti/TiN and Ti/TiO_2_-N samples, the slope of the diagram is reduced almost in the same way, converging 1593 HV_0.5_ at a distance of 15 microns from the surface. The higher hardness of the Ti/TiO_2_-N sample is the presence of the PEO oxide intermediate layer, which makes the hardness of about 215 Vickers higher than the Ti/TiN sample. The hardness was increased by three times for the Ti/TiO_2_ sample due to thicker coating, while the hardness of the PEO coating was more than doubled by nitriding.

Overall, it may be said that the high concentration of active nitrogen species increases the number of nitride phases in the composite layer and the formation of interstitial solid solutions in the diffusion zone [3]; therefore, the hardness compared to uncoated titanium is increased by about six times. The surface hardness in the two-stage process is higher than that of the other two processes, so that the slope of the cross-section hardness changes in the two-stage process does not change significantly compared to the gas nitriding, and it is remarkably improved compared to the PEO process due to less hardness of the oxide phases in comparison to nitride phases. The presence of supersaturated nitrogen (nitriding process leads to the formation of the nitrogen supersaturated α titanium phase) makes some distortion in the lattice and significantly increases the hardness. In other words, the high amount of nitrogen in the network and the formation of nitride precipitates enhance the hardness level [41]. Moreover, the formed nanocrystallites in the structure and closure and shrinkage of pores in the porous layer positively affect the hardness of the Ti/TiO_2_-N sample.

### 3.4. Electrochemical Behavior of the Coating

**Figure 8:**
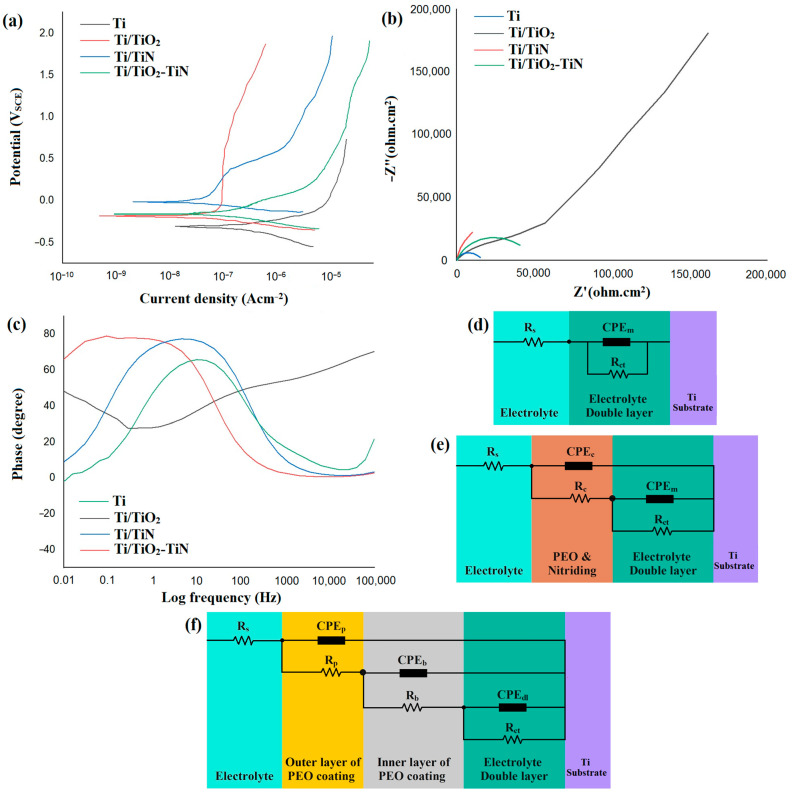
(**a**) Potentiodynamic polarization curves. (**b**) Nyquist and (**c**) Bode phase diagrams and proposed equivalent circuit for EIS results of bare and coated samples; (**d**) Ti and Ti/TiN; (**e**) Ti/TiO_2_-N; and (**f**) Ti/TiO_2_.

Figure 8a show the potentiodynamic polarization curves and the polarization data, respectively. This test was performed to compare the corrosion behavior of uncoated and coated specimens in 3.5% sodium chloride solution after 30 min of immersion. The cathodic branch of the studied curves’ slope shows that their slope changes are Ti/TiN > Ti > Ti/TiO_2_-N > Ti/TiO_2_. Increasing the slope indicates that the corrosion is under the control of the cathodic reactions because, in the cathodic controlling mechanism, the corrosion potential becomes more active, and the corrosion rate is increased [42,43,44,45,46,47]. The corrosion potential (E_corr_) of the uncoated Ti, Ti/TiO_2_, and Ti/TiO_2_-N coated specimens was −0.382 V_SCE_, −0.011 V_SCE_, and −0.231 V_SCE_, respectively. The corrosion current density (i_corr_) of the uncoated Ti, Ti/TiO_2_, and Ti/TiO_2_-N coated specimens was 254, 12, and 33 nA/cm^2^, respectively. For the coated samples, the first effect is that the anodic branch of the curves is shifted to the left, but due to the presence of pinholes and cracks, the surface of the coating is subjected to corrosive ion attack, and the anodic branches break down and tend to the high current density. The slopes of the anodic branch for the coatings and the uncoated sample have a significant change, which can be explained in the case of the uncoated sample starting to passivate immediately after polarization and at a current density of about 10^−5^ A·cm^–2^, which forms a protective film on the metal surface. Changes to the anodic slope for Ti/TiN sample include three stages: in the first stage and beginning of the anodic zone, the surface is slightly corroded, and then, in the second stage, the surface is passivated, and at potential 1.5 V this protective layer is broken down (third stage). The formation of a protective oxide layer on Ti/TiN is due to the penetration of chloride ions into the cavities and surface cracks of this coating [42,43]. After contact with the metal/coating interface region’s metal surface, it slightly corrodes the metal surface. Ultimately, due to Ti’s active/passive behavior, this area has become passivated and has created a second area that continues until the breakdown at higher potentials. All samples show the active zone at the beginning of their anodic branch where the current density increases rapidly. This rapid increase in the active zone is mainly due to the metal oxidation process in the metal/oxide interface, and a weaker passive layer is formed again [48]. The difference between the anodic branches of Ti/TiO_2_ and Ti/TiO_2_-N samples is due to nitrogen in the oxide coating structure. In general, it can be said that the initial corrosion rate in Ti/TiO_2_-N sample is lower than in Ti/TiO_2_ sample and behaves as a Ti/TiN sample immediately after starting the anodic branch. However, due to favorable reactions regarding the presence of nitrogen in TiO_2_, this film is broken and re-oxidized at a higher potential and begins to passivate. In other words, the Ti/TiO_2_-N sample exhibits a behavior in-between the Ti/TiO_2_ and Ti/TiN samples.Generally, when the passive critical current density is low, the protective layer is easily formed, and the lower the passive current density and the higher the transpassive potential, the more protective the layer will be [48].

The results show that the corrosion potential increases as a criterion of the thermodynamic tendency of the coating to corrode compared to the uncoated sample, which means more chemical stability and less corrosion tendency. In other words, the corrosion potential of the coatings has also shifted to more positive values than the corrosion potential of the uncoated sample, indicating anodic control of the corrosion process by these coatings. The corrosion potential of the samples has been changed to Ti/TiO_2_ > Ti/TiO_2_-N > Ti/TiN > Ti; this means that the tendency of the Ti/TiO_2_ sample to corrode is less than the Ti/TiN and Ti/TiO_2_-N, possibly due to the high nitrogen penetration into the titanium oxide lattice. The presence of nitrogen in the TiO_2_ lattice caused the coating to be electrically conductive and transformed from an insulating material to a semiconductor, which resulted in higher reactivity (electron transfer and charge exchange) at the coating surface. Ultimately, the corrosion rate has been increased. Significant slope changes have occurred in the samples, wherein the turning point in the potential implies an alteration in surface film formation kinetics. The corrosion current density was obtained by Tafel extrapolation by the impact of tangent lines on the linear region of the anodic and cathodic branches. The shift in corrosion current density of the coated samples indicates a limited anodic reaction and higher chemical stability than titanium. In other words, the decrease in current density indicates a decrease in the rate of electrochemical reactions or the inhibition of corrosion reactions. It is observed that the corrosion current density for Ti/TiO_2_,, Ti/TiO_2_-N, and Ti/TiN decreased significantly compared to the uncoated Ti sample, respectively.

The polarization resistance (*R_p_*) is calculated based on the linear polarization behavior near the open circuit potential or Tafel extrapolation. The linear *R_p_*, which is inversely proportional to the value of the *i_corr_*, is as follows [15]:

(3) $$R_{p}=\frac{\beta_{a}\beta_{c}}{2.3(\beta_{a}+\beta_{c})i_{corr}}\;$$

The polarization resistance of the Ti/TiO_2_-N and Ti/TiO_2_ coatings is 632.2 and 1451.9 kΩ cm^2^, respectively. These values are significantly higher than that of the uncoated Ti (135.9 kΩ cm^2^) and Ti/TiN coated sample (275.6 kΩ cm^2^). In general, coatings can increase corrosion resistance by increasing the charge transfer resistance in the metal–electrolyte interface, limiting the absorption of aggressive ions and increasing the substrate potential [49]. It can be said that, with increasing thickness and compactness, the TiO_2_ coating restricts the penetration of the corrosive solution to the substrate, and the corrosion resistance is improved. The Ti/TiN sample coating also increases the polarization resistance of titanium by forming a nitride protective layer, but it is less resistant due to being more active than titanium oxide. Ti/TiO_2_-N duplex coating has resistance between Ti/TiO_2_ and Ti/TiN samples because it has intermediate behavior. The formation of oxide coating on titanium reduces the active surface area for corrosion mechanisms, but the presence of added nitrogen in the structure increases the reactivity of this sample to exposure to corrosive ions and reduces corrosion resistance. The corrosion rate of the samples was calculated, and it was found that the lowest corrosion rate was related to the Ti/TiO_2_ and Ti/TiO_2_-N samples, which are significantly lower compared to the uncoated Ti sample.

Figure 8b,c illustrates the Nyquist and Bode phase diagrams for uncoated and coated samples after 30 min of immersion in 3.5% NaCl. The equivalent circuit in Figure 8d–f was employed to fit EIS data for uncoated Ti, Ti/TiN, Ti/TiO_2_, and Ti/TiO_2_-N samples. The corrosion resistance mechanism of the coatings was investigated by electrochemical impedance spectroscopy, which is a powerful method to evaluate the corrosion behavior of the coatings. The Nyquist curves for the uncoated titanium substrate and TiN coating have a semicircle capacitive loop at all frequencies. This behavior has represented the formation of a dielectric layer on the surface of the maples and the presence of a time constant in the equivalent circuit. Corrosion resistance was obtained by measuring the semicircle diameter in the Nyquist curves. In contrast, the Nyquist curve of the sample with a TiO_2_ coating has three capacitive semicircles. The larger semicircle appearing at lower frequencies is related to the dense inner layer, and the metal/coating interface and the smaller semicircle at higher frequencies are attributed to the outer porous layer. The dense internal layer is formed due to the applied voltage on the metal, and the porous layer is formed due to the reaction of the plasma with the metal and the coating bath during the process [2,50,51]. The Nyquist curve of Ti/TiO_2_-N has two time-constant elements in the proposed equivalent circuit, and the smaller semicircle at high frequencies corresponds to the outer porous nitrided layer, and the larger semicircle at lower frequencies is attributed to the outside dense TiO_2_ coating and the double layer. Generally, the high-frequency capacitive behavior reflects the coating activity, and indeed, its increase indicates the saturation of the coating from the corrosive product. The low-frequency loop shows the charge transfer reactions and the capacitive nature of the dielectric double layer.

In the proposed equivalent circuits, R_S_ represents the solution resistance between the working electrode and the reference electrode; R_ct_, R_c_, R_p,_ and R_b_, are charge transfer resistance, nitride layer resistance, external porous layer resistance, and internal dense layer resistance, respectively. Due to the heterogeneities of the nitride, porous, and dense layers for the coated samples and the uncoated sample surface roughness, the electrochemical reactions of these layers have been characterized by the definition of constant phase element (CPE). The CPE is used instead of the ideal capacitor with a different impedance definition. Considering that resistance is inversely relative to capacitance when R_pore_ rises and CPE-coat diminishes, the coating barrier feature escalates, and its protective effectiveness elevates. The double layers (CPEdl–T) of the uncoated Ti, Ti/TiN, Ti/TiO_2_-N, and Ti/TiO_2_ samples were 33.23, 10.07, 0.76, and 0.18 µF/cm^2^, respectively. The capacitance impedance is Z = 1/jωc, and this the value for CPE is Z = 1/(Y_0_jω)^n^, where C stands for capacitance, ω for phase, Y_0_ for admittance (inverse of the impedance and the equivalent to the capacitance in an ideal capacitor), and j is a unit imaginary number. It can be seen that the difference between the CPE and C is only at a power n, which is a numerical value between zero and one, with zero being the ideal resistor and one being the ideal capacitor [38].

Regarding Bode phase charts obtained from the EIS test, the diagrams comprise two distinct forms: phase changes versus frequency logarithms. The maximum phase angle shift to the right is evident in the curve of the Ti/TiO_2_ compared to the other samples. The maximum point on the diagram shifts to the right, and this indicates the higher corrosion protection properties of the surface. The Ti/TiO_2_ sample created the highest Bode phase angle of −79°, whereas lower maximum phase angles of −62° were observed for the uncoated Ti substrates, which implies their lower corrosion resistance. Since TiO_2_ film generated by PEO approaches acted as a barrier to prevent electrolyte solution diffusion to the interface of magnesium and the tortuosity pathway for the diffusion of electrolyte increased, thus the substrate corrosion rate was reduced [8]. These results demonstrated that the TiO_2_ coating could appropriately delay the penetration of the corrosive solution and work as a solid barrier towards corrosion.

In the electrochemical impedance diagrams, the increase in capacitance values indicates the entrance of ions and corrosive solution into the coating and generally a decrease in coating resistance, accompanied by a decrease in the resistance of these elements. The coated specimens show higher resistance than the uncoated substrate, which indicates higher corrosion resistance. The TiO_2_ coating leads to a significant escalation in the charge transfer resistance (R_ct_) of the uncoated and coated Ti substrate. Thus, the Rct of samples is as follows: uncoated Ti (12.89 kΩ m^2^) < Ti/TiN (18.14 kΩ m^2^) < Ti/TiO_2_-N (42.16 kΩ m^2^) < Ti/TiO_2_ (168.36 kΩ m^2^). Additionally, both dense inner and outer porous layers have significantly lower capacitance, this indicates higher resistance to corrosion of the PEO and duplex coated specimens. The lower that the capacitance is charged, the less the current. When a capacitor is omitted from the circuit, no current can flow because, when the capacitor is charged, the current will not pass, and the circuit will be cut off. The dense oxide layer on the surface of the titanium also has this effect and, such as a capacitor after being fully charge, disrupts the electrical circuit by reducing the corrosion rate [52,53,54,55,56,57,58,59,60,61,62,63,64,65,66,67,68,69,70]. Based on the EIS diagrams and the obtained data, it can be concluded that the corrosion resistance of the sample with plasma electrolytic oxidation coating is better than that of the uncoated sample. In general, for Ti/TiO_2_ and Ti/TiO_2_-N, it can be said that, due to the easy penetration of the corrosive solution into the outer layer cavities, this layer did not have much effect on the electrochemical impedance spectrum. Therefore, this layer has no role in corrosion resistance. It is, therefore, reasonable to accept the inner layer of the coating is a barrier against corrosion attacks. This layer creates a physical barrier against corrosive solution and increases corrosion resistance.

## 4. Conclusions

The gas nitriding process on the surface of titanium and titanium oxide revealed that nitrogen penetrated the surface of the samples and formed TiN and TiO_0.34_N_0.74_ phases. FTIR analysis was also performed to confirm the X-ray diffraction analysis and showed that nitrogen–oxygen bonds were formed in the nitrated titanium oxide sample. Morphological analysis of gas nitrided titanium and duplex gas nitrided and PEO showed that the first structure has a dense appearance with surface pits. In contrast, the second structure is similar to the titania-coated sample by PEO except that some spherical particles have emerged near the cavities. Examination of the wear behavior of the specimens showed that the coefficient friction improved, and the abrasion resistance highly increased, possibly due to the increased hardness of the coating. The corrosion behavior of Ti/TiO_2_, Ti/TiN, and Ti/TiO_2_-N coated specimens showed that the highest corrosion resistance was for the Ti/TiO_2_ because of the barrier effect of the coating and electrical charge transfer resistance. These findings verify that the combination of PEO and gas nitriding methods is very effective on corrosion and wear properties. Therefore, different structures, thicknesses, and surface properties are generated. This approach exhibited that the corrosion behavior and wear resistance of Ti was enhanced substantially. Suggesting the creation of novel coating systems with productive corrosion and wear performance, besides presenting self-healing characteristics, also permit additional corrosion protection and further wear resistance, which are vital to expanding the application of Ti and its alloys.

## Figures and Tables

**Table 1:** The crystalline properties of the coatings.

Sample	FWHM	2θ_max_	Average Theoretical Crystallite Size (nm)
	(Degree)	(Degree)	
Ti/TiO_2_	0.3444	35.91	24
Ti/TiN	0.3444	36.11	27
Ti/TiO_2_-N	0.2952	7.36	22

## Data Availability

All data provided in the present manuscript are available to whom it may concern.

## Supplementary Materials

The following supporting information can be downloaded at: https://www.mdpi.com/article/10.3390/ma15238300/s1, Table S1: Chemical composition of titanium as substrate.
