# Supplementary material for: Corrosion and Wear Behavior of TiO2/TiN Duplex Coatings on Titanium by Plasma Electrolytic Oxidation and Gas Nitriding
Source: Materials (Basel). 2022 Nov 22;15(23):8300. doi: 10.3390/ma15238300 (PMC9741034; doi:10.3390/ma15238300)
Supplement: Supplementary file 1 [file materials-15-08300-s001.zip › materials-1882236-Table S1.pdf]

Table S1. Chemical composition of titanium as substrate.

Element	Ti	Fe	C	N	O	Other
Concentration (wt. %)	99.34	0.13	0.009	0.014	0.01	0.497
